# Human proteins that interact with RNA/DNA hybrids

**DOI:** 10.1101/gr.237362.118

**Published:** 2018-09

**Authors:** Isabel X. Wang, Christopher Grunseich, Jennifer Fox, Joshua Burdick, Zhengwei Zhu, Niema Ravazian, Markus Hafner, Vivian G. Cheung

**Affiliations:** 1 Howard Hughes Medical Institute, Chevy Chase, Maryland 20815, USA;; 2 Life Sciences Institute, University of Michigan, Ann Arbor, Michigan 48109, USA;; 3 Neurogenetics Branch, National Institute of Neurological Disorders and Stroke, NIH, Bethesda, Maryland 20892, USA;; 4 Department of Pediatrics, University of Michigan, Ann Arbor, Michigan 48109, USA;; 5 Laboratory of Muscle Stem Cells and Gene Regulation, National Institute of Arthritis and Musculoskeletal and Skin Diseases, Bethesda, Maryland 20892, USA

## Abstract

RNA/DNA hybrids form when RNA hybridizes with its template DNA generating a three-stranded structure known as the R-loop. Knowledge of how they form and resolve, as well as their functional roles, is limited. Here, by pull-down assays followed by mass spectrometry, we identified 803 proteins that bind to RNA/DNA hybrids. Because these proteins were identified using in vitro assays, we confirmed that they bind to R-loops in vivo. They include proteins that are involved in a variety of functions, including most steps of RNA processing. The proteins are enriched for K homology (KH) and helicase domains. Among them, more than 300 proteins preferred binding to hybrids than double-stranded DNA. These proteins serve as starting points for mechanistic studies to elucidate what RNA/DNA hybrids regulate and how they are regulated.

RNA/DNA hybrids are abundant in human cells. They form during transcription when nascent RNA is in close proximity to its DNA template. The resulting RNA/DNA hybrids and the displaced single-stranded (ss) DNA are called R-loops. RNA/DNA hybrids are structurally different and more stable than the corresponding double-stranded DNAs (Bhattacharyya et al. 1990; Roberts and Crothers 1992).

RNA/DNA hybrids are found in origins of replication (Baker and Kornberg 1988; Xu and Clayton 1996), immunoglobulin class-switch regions (Yu et al. 2003), and transcription complexes (Hanna and Meares 1983; Nudler et al. 1997; Skourti-Stathaki et al. 2011). R-loops were mostly viewed as deleterious because they can lead to DNA damage. The unpaired DNA strand is vulnerable to damage (Huertas and Aguilera 2003; Li and Manley 2005; Mischo et al. 2011; Wahba et al. 2011), and improper processing of R-loops such as those mediated by transcription-coupled excision repair also results in DNA damage (Sollier et al. 2014). Increasingly, studies have shown that R-loops have regulatory roles. They are found abundantly in human gene promoters and terminators where RNA processing takes place (Ginno et al. 2012; Chen et al. 2017). Given these opposite impacts of R-loops, their formation and resolution must be regulated tightly. Genome-wide methods have mapped and quantified R-loops in yeast to human cells (Chan et al. 2014; El Hage et al. 2014; Wahba et al. 2016; Chen et al. 2017). With these methods, studies have shown that too many and too few R-loops lead to pathologic consequences. In immunodeficiencies such as Wiskott–Aldrich syndrome (Sarkar et al. 2017), and neurodegenerative diseases such as Friedreich ataxia (Groh et al. 2014), patients have more R-loops, whereas cells from ALS4 patients with the senataxin mutation have fewer R-loops (Grunseich et al. 2018).

Because the number and location of hybrids are critical to maintaining cellular function, most likely there are regulatory proteins that distinguish RNA/DNA hybrids from their double-stranded (ds) DNA counterparts. The structures of RNA/DNA hybrids with different sequences have been studied alone (Benevides et al. 1986; Fedoroff et al. 1993) and in complex with different proteins (Rychlik et al. 2010; Figiel and Nowotny 2014; Nishimasu et al. 2014; Bernecky et al. 2016). The results show that RNA/DNA hybrids do not adopt the traditional B-conformation of DNA or A-conformation of RNA but occur as mixtures or heteromerous duplexes (Fedoroff et al. 1993). It is well known that regulatory proteins recognize their targets by nucleic acid sequences and/or structures. Transcription factors often identify their targets based on sequences. In contrast, there are proteins that recognize their targets by structures and not just by sequences. Some proteins can target specifically different components (the RNA or hybrid) of the R-loops. For example, the conformation of R-loop is critical for the cleavage of the two DNA strands by Cas9 (Jiang et al. 2016). Ribonuclease H1 (also known as RNase H1) cleaves the RNA of RNA/DNA hybrids (Fedoroff et al. 1993; Cerritelli and Crouch 1995), whereas activation-induced cytidine deaminase (AID) favors binding to RNA/DNA hybrids (Abdouni et al. 2018). Recently, we showed that DNA methyltransferase 1 (DNMT1) binds more avidly to dsDNA than to the corresponding RNA/DNA hybrids; thus, the formation of the hybrid promotes transcription by preventing methylation-induced silencing (Grunseich et al. 2018). Presumably, there are other proteins like DNMT1 whose regulatory roles can be influenced by RNA/DNA hybrids.

The roles of RNA/DNA hybrids are beginning to be recognized, but much remains unknown. It is not clear what regulates the formation and resolution of RNA/DNA hybrids. It is also not known how hybrids affect processing of RNA and what transcriptional steps they regulate. Naturally occurring mutations and yeast mutant collections have facilitated much of the mechanistic studies of R-loops. But the mutant screens alone have yet to yield a comprehensive view of R-loops. High-throughput methods to identify proteins that interact with nucleic acids have provided valuable information on gene regulation (Hafner et al. 2010; Zhao et al. 2010; Baltz et al. 2012; Panda et al. 2014). A comprehensive list of proteins that interact with R-loops will facilitate studies on formation and processing of R-loops as well as their regulatory roles. Here, we report pull-down assays followed by mass spectrometry and in vivo confirmation studies that identified more than 800 proteins that bind to the RNA/DNA hybrids of R-loops in human cells.

## Results

**Figure 1.**
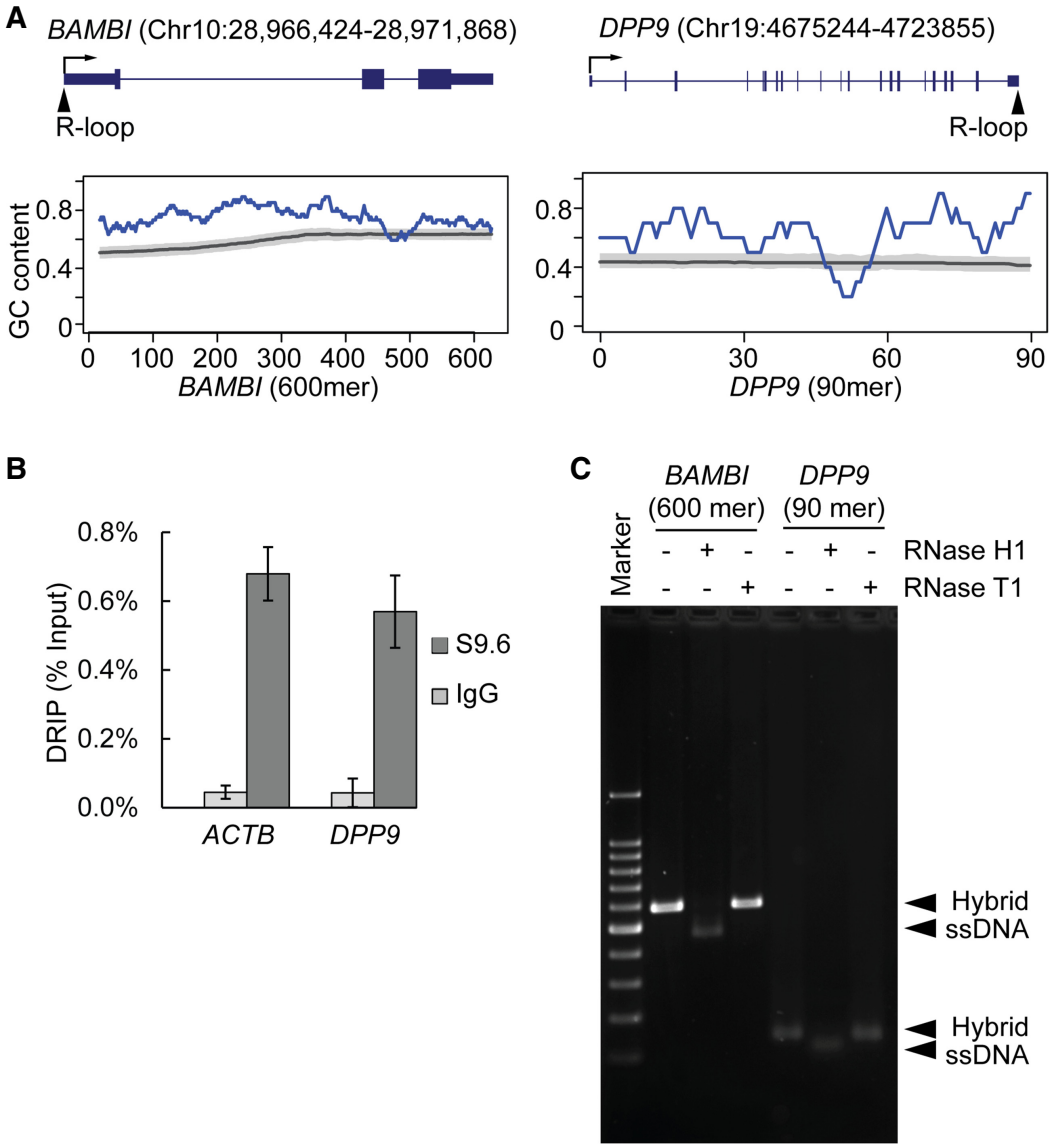
R-loops at GC-rich regions in the *BAMBI* promoter and *DPP9* 3′ UTR. (*A*) Location and GC content of sequences underlying R-loops in *BAMBI* and *DPP9*. (*Top*) R-loop location is marked on gene models of *BAMBI* and *DPP9*. Boxes represent exons, and lines represent introns. Arrows show transcription start site and direction of transcription. (*Bottom*) GC content of the 600-mer and 90-mer sequences corresponding to RNA sequence in the R-loops (blue line). GC content is calculated as (G + C)/(G + C + A + U) in the 50-nt sliding window for 600-mer or 10-nt sliding window for 90-mer. Genome background of GC content is calculated from corresponding regions of 14,587 RefSeq genes that are at least 2 kb long and 1 kb away from neighboring genes. The gray line represents median GC content, and the shade represents ±10%. (*B*) The S9.6 antibody specifically pulled down R-loops at the 3′ UTR of *DPP9*. DRIP was carried out using an S9.6 antibody or nonspecific IgG. Precipitated DNA was amplified using primers specific for *DPP9* 3′ UTR. Primers specific for a previously reported R-loop region at the 3′ UTR of *ACTB* were used as positive control. (Error bars) SEM of triplicates. (*C*) Integrity of the RNA/DNA hybrid was confirmed using RNase H1 and RNase T1. As expected, RNase H1 specifically digested RNA in the hybrids, leaving ssDNA as a product. RNase T1, which is specific for ssRNA, did not cleave the hybrids.

To identify proteins that bind to RNA/DNA hybrids, we made hybrids corresponding to two R-loops identified previously by S9.6 DRIP-seq (Grunseich et al. 2018): One is in the 5′ end of the *BAMBI* gene, and the second is in the 3′ end of the *DPP9* gene. We synthesized 600-mer and 90-mer RNA/DNA hybrids that correspond to sequences underlying R-loops in *BAMBI* and *DPP9*, respectively. Figure 1A shows the locations of the R-loops in *BAMBI* and *DPP9*. The *BAMBI* and *DPP9* regions are GC-rich, with GC content of 76% and 62%, respectively, consistent with findings that regions with G-rich RNA and complementary C-rich DNA are prone to hybrid formation (Roy and Lieber 2009; Skourti-Stathaki et al. 2011; Ginno et al. 2012). The R-loop in the *BAMBI* promoter was extensively characterized previously (Grunseich et al. 2018). We validated the R-loop in the 3′ UTR of *DPP9* by S9.6 precipitation in this study (Fig. 1B). To check the integrity of the two RNA/DNA hybrids, we confirmed their sensitivity to RNase H1 (Cerritelli and Crouch 1995, 2009) and resistance to ribonuclease T1 (rntA, also known as RNase T1) (Fig. 1C; Zuo and Deutscher 2002).

**Figure 2.**
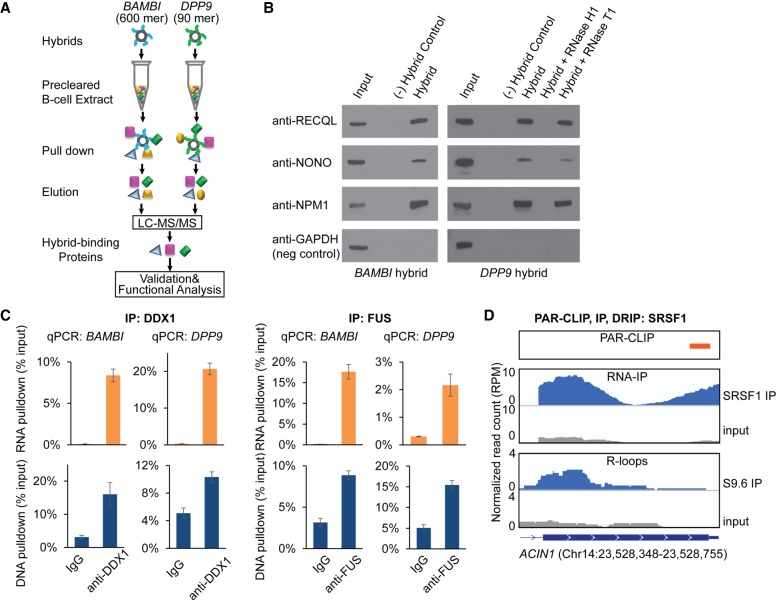
Identification of proteins that bind specifically to RNA/DNA hybrids. (*A*) Schematic of the experimental procedure. Biotinylated hybrids were conjugated to streptavidin beads and incubated with B-cell extracts. The proteins pulled down by hybrids were identified through proteomic analysis (LC-MS/MS). Only proteins that were bound by both hybrids were retained for further analysis. (*B*) Hybrid-binding proteins identified by proteomic analysis were validated by Western blot. B-cell extract (input) was incubated with no hybrid as a negative control, *BAMBI* or *DPP9* hybrid, *DPP9* hybrid pretreated with RNase H1 or RNase T1, respectively. Proteins were pulled down by biotinylated hybrids and analyzed by Western blot. Interactions with the pulled down proteins were eliminated by RNase H1 digestion but were not affected by RNase T1 digestion. (*C*) Validation of protein-hybrid interaction by reverse pull down. DDX1 and FUS and their associated hybrids were pulled down by anti-DDX1 and anti-FUS antibodies, respectively. RNA and DNA were purified from precipitates and quantified by qPCR using primers annealing to *DPP9* or *BAMBI* hybrids. Input amount was normalized against copy numbers of DNA and transcripts. (Error bars) SEM of triplicates. (*D*) Colocalization of SRSF1 binding with R-loops. SRSF1 binding to the *ACIN1* transcript was identified by PAR-CLIP and RNA-IP using an anti-SRSF1 antibody, and R-loops were identified by S9.6 DRIP. IGV viewer screenshots of data showing sequence reads from PAR-CLIP, RNA-IP, and DRIP-seq.

To find proteins that bind to these hybrids, we added biotinylated forms of the *BAMBI* and *DPP9* hybrids to human B-cell extracts and carried out pull-down assays (Fig. 2A). Liquid chromatography followed by tandem mass spectrometry (LC-MS/MS) were performed to identify the proteins bound to the two hybrids. We used stringent inclusion criteria (Methods); each protein must be represented by four or more peptides with unique sequences. Despite these criteria, we identified a large number of proteins in the pull-down assays, namely, 1460 proteins with the *BAMBI* hybrid and 1018 proteins with the *DPP9* hybrid, in which 803 proteins were identified by both (Supplemental Table S1). Among the proteins identified in our *BAMBI* and *DPP9* pull-down assays are RNase H1 (RNASEH1) and XRN2, which are known to bind to and modify RNA/DNA hybrids (Table 1), confirming that our approach identifies enzymes that process RNA/DNA hybrids (Stein and Hausen 1969; Keller and Crouch 1972; Skourti-Stathaki et al. 2011). Most of the identified proteins have not been reported to associate with hybrids. We validated the interaction between proteins and hybrids by Western blot (Fig. 2B). To ensure that the proteins are binding to hybrids and not to single-stranded RNAs, we showed that digestion by RNase H1 abolished the interactions, whereas RNase T1 did not interfere with the protein-hybrid interactions.

**Table 1.**
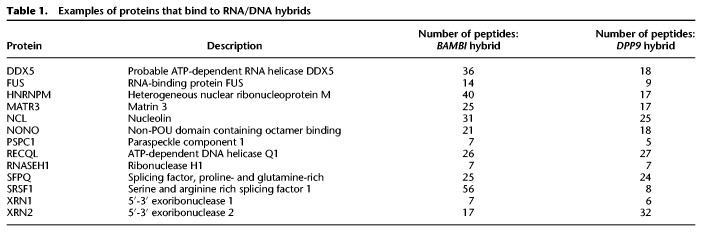
Examples of proteins that bind to RNA/DNA hybrids

Many of the hybrid-binding proteins interact with R-loops in human cells. To ensure that the hybrid-interacting proteins we identified reflect in vivo interactions, we carried out three independent analyses. First, we looked for overlap between our proteins and those determined by Gromak and colleagues to bind to R-loops immunoprecipitated from HeLa cells with the S9.6 antibody (Cristini et al. 2018). Although their study was performed using HeLa cells and our study was carried out with human B-cell extracts, 197 of the proteins identified in their study were also found in our study (Supplemental Table S1). This provides evidence that the hybrid-binding proteins we identified interact with R-loops in vivo. Second, we validated the hybrid-protein interaction by reverse immunoprecipitation. Using antibodies specific for DDX1 and FUS, we pulled down the protein-nucleic acid complexes, then by quantitative PCR, we showed enrichment of DNA and RNA corresponding to the *BAMBI* and *DPP9* hybrids (Fig. 2C). Thus, the results validate that DDX and FUS bind in vivo to *BAMBI* and *DPP9* hybrids. Third, we assessed globally the binding of SRSF1, one of the hybrid-binding proteins, to R-loops in vivo. SRSF1 is a member of the serine/arginine-rich splicing factors that binds to exon-splicing enhancers (Pandit et al. 2013). We identified transcripts bound by SRSF1 using two independent methods: PAR-CLIP and RNA-IP. Then we characterized R-loop regions in human cells using DRIP-seq with the S9.6 antibody. We carried out this experiment to assess the number of R-loops with which these hybrid-binding proteins interact. Given the large number of hybrid-binding proteins, each can be interacting with a few or many R-loops. Here, we began by addressing one protein. The results showed that SRSF1 binds to *BAMBI*, *DPP9*, and >20% of R-loops in human B-cells. Figure 2D shows an example of the colocalization of R-loops and SRSF1 binding sites in *ACIN1*. Together, these results support that the hybrid-binding proteins we identified bind to many R-loops in human cells, in addition to the *BAMBI* and *DPP9* hybrids.

The hybrid-binding proteins, such as FUS, matrin 3, and ligase 3, have significant enrichment of domains that bind nucleic acids and participate in a broad spectrum of gene regulation. A search of domains found in the hybrid-binding proteins reveals that 50 have alpha-beta plait and 27 contain OB-fold. A helicase domain, such as that in AQR that resolves hybrids, is also found (Sollier et al. 2014). Examples of the functional domains that are highly enriched in the hybrid-binding proteins are listed in Table 2. Among these 803 hybrid-binding proteins, 354 have disordered protein domains, including 59 proteins with [G/S]Y[G/S] amino acid motif that is hydrophobic and confers the proteins the ability to form hydrogels (Supplemental Table S1; Casas-Finet et al. 1993; Frey et al. 2006). Disordered regions provide protein flexibility in structure and function. In the hybrid-binding proteins, these regions likely allow the proteins to scan for target structures and interact with a range of other proteins and nucleic acid targets (Oldfield and Dunker 2014). The 803 hybrid-binding proteins cover a range of functions. Table 3 shows five functional categories that are highly enriched with hybrid-binding proteins. It shows that these proteins participate in multiple RNA processing steps, including splicing, pre-mRNA processing, and unwinding of RNA. The hybrid-binding proteins include PABPC1 (Kuhn et al. 2003) and CPSF1 (Murthy and Manley 1995) that bind poly(A) sequences, which is somewhat unexpected considering the absence of polyadenine track in our hybrids. Therefore, these proteins may be recognizing structures that are shared by poly(A) RNA and the hybrids.

**Table 2.**
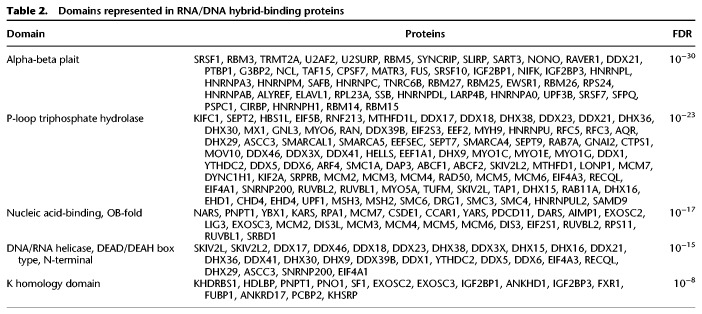
Domains represented in RNA/DNA hybrid-binding proteins

**Table 3.**
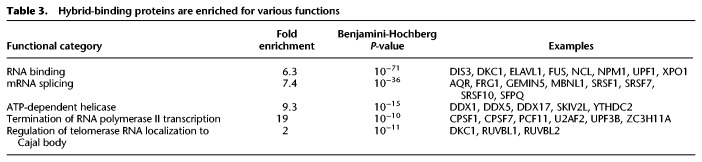
Hybrid-binding proteins are enriched for various functions

The *BAMBI* and *DPP9* hybrids pulled down several protein complexes. These include the *Drosophila* behavior/human splicing (DBHS) complex that comprises the non-POU domain containing octamer binding protein (NONO) and paraspeckle protein component 1 (PSPC1). DBHS proteins form heterodimer and oligomers with multiple domains for RNA binding (Passon et al. 2011, 2012). The resulting combinations likely provide surfaces that facilitate binding to RNA/DNA hybrids. Other protein complexes include RPA1, RNase H1 (Nguyen et al. 2017), and THRAP3/BCLAF1 (Vohhodina et al. 2017). Thus, our pull-down assays identify direct RNA-protein and indirect RNA-protein interactions mediated by protein–protein interactions.

Next, we studied the RNA/DNA hybrid-binding proteins to look for those that are repelled or attracted by hybrids relative to other nucleic acid structures. RNA/DNA hybrids are formed during transcription when nascent RNA hybridizes with their template DNA, thus disrupting the dsDNA. In a previous study, we showed that hybrid formation deters methylation-dependent gene silencing because DNA methyltransferase 1 binds less avidly to RNA/DNA hybrid than dsDNA (Grunseich et al. 2018). We assume that there are other proteins that are repelled by hybrids or attracted to them. To look for these proteins, we repeated the pull-down assays with dsDNA from the *BAMBI* and *DPP9* regions. We then compared the proteins that were pulled down by the nucleic acids of the same sequences but with different structures, that is, hybrids versus dsDNA. We found proteins like DNMT1 that bind more avidly to dsDNA than the RNA/DNA hybrids. Confirming our previous study that showed DNMT1 is repelled by hybrids, we found DNMT1 bound to both hybrids and the corresponding dsDNA, but dsDNA forms of *BAMBI* and *DPP9* pulled down more DNMT1 than their hybrids. With these results, we looked for similar binding patterns in other proteins and found 84 other candidates, such as PARP1 and UHRF1, that are repelled by hybrids (Supplemental Table S2). The ubiquitin transferase, UHRF1, interacts with and recruits DNMT1 (Sharif et al. 2007); thus, it could be repelled because it complexes with DNMT1. PARP1 can also be part of a complex with UHRF1 and DNMT1 (Sharif et al. 2007), and it regulates UHRF1's interaction with DNMT1 by addition of poly(ADP-ribose) (De Vos et al. 2014). In addition, PARP1 binds gene promoters and regulates transcription (Krishnakumar et al. 2008). Because hybrids disrupt binding of PARP1, their formation can affect PARP1 and lead to direct and indirect transcriptional consequences. Although PARP1 is mostly studied as a DNA-binding protein, a recent study showed that PARP1 binds RNA, in particular, those that are GC-rich (Melikishvili et al. 2017). Thus, it is possible that PARP1 recognizes non-B nucleic acid structures that are shared between GC-rich RNA and RNA/DNA hybrids.

**Figure 3.**
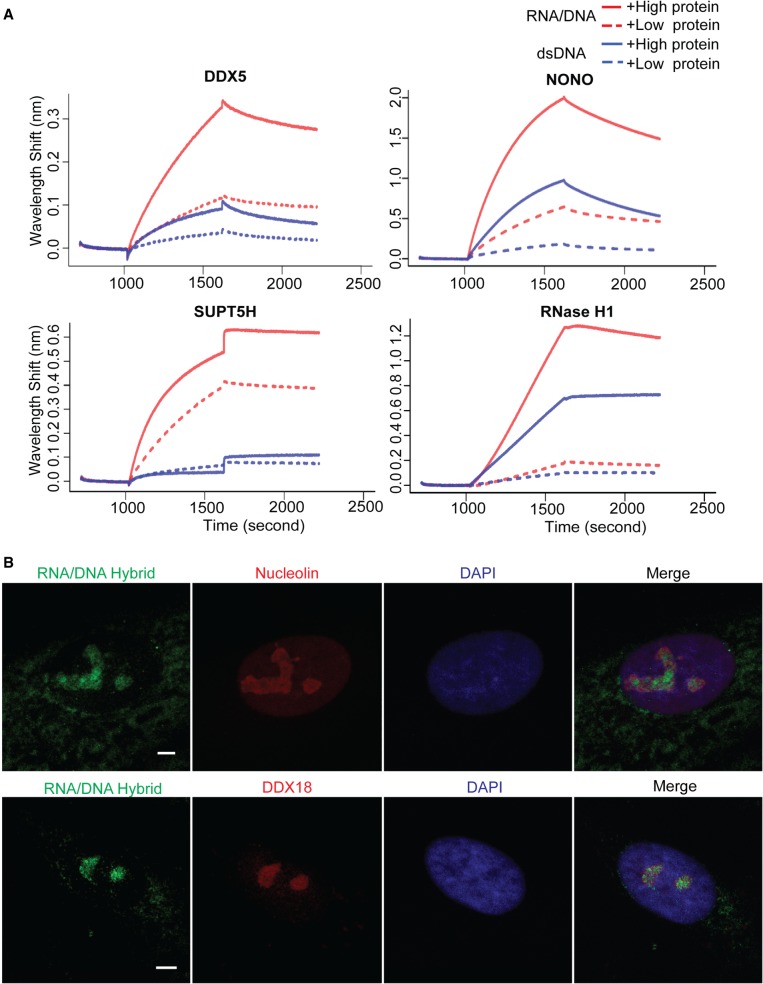
Validation of protein and hybrid interaction. (*A*) The protein and hybrid interaction is shown by biolayer interferometry. Binding of *DPP9* RNA/DNA hybrid (red lines) or dsDNA (blue lines) to high concentration (solid lines) or low concentration (dotted lines) of each protein was measured. These proteins showed more avid binding to RNA/DNA hybrid than to dsDNA. Baseline was recorded from 0 to 1000 sec, association of protein with dsDNA or RNA/DNA hybrid from 1000 to 1600 sec, followed by dissociation. (*B*) Immunofluorescence staining in primary human fibroblasts showing colocalization of nucleolin and DDX18 (red) with the R-loops stained by S9.6 RNA/DNA hybrid antibody (green). DAPI staining is in blue. (Scale bar) 1 µm.

In addition to proteins that are repelled by hybrids, there are proteins that are attracted by RNA/DNA hybrids. We found 364 proteins that are attracted by both *BAMBI* and *DPP9* hybrids compared to corresponding dsDNA (Supplemental Table S3). There are 14 proteins that bound to only the hybrid forms of *BAMBI* and *DPP9* but not to the corresponding dsDNA. These include several members of the nuclear exosomes such as DIS3L, EXOSC3, and EXOSC6. In addition, there are 350 proteins that favor the hybrid forms of *BAMBI* and *DPP9* compared to the corresponding dsDNA. We validated the affinity of some of these proteins to RNA/DNA hybrids versus dsDNA by biolayer interferometry. Figure 3A shows that DDX5, NONO, SUPT5H, and RNase H1 (RNASEH1) have a higher affinity for RNA/DNA hybrids than the corresponding dsDNA. These hybrid-binding proteins are significantly enriched for K homology (KH) domains and RNA/DNA helicase. Immunostaining of human cells with S9.6 antibody confirmed colocalization of the nuclear RNA/DNA hybrids with proteins that were identified to be attracted by RNA/DNA hybrid, nucleolin (NCL), and DDX18 (Fig. 3B). There are 24 RNA/DNA helicases and 12 proteins with KH domains. Because the sequences of the *BAMBI* and *DPP9* hybrids are distinct, the proteins that bind to both of them likely recognize their hybrid structures rather than sequences. Some of these hybrid-binding proteins, such as nucleolin (González et al. 2009; Haeusler et al. 2014) and FUS (Takahama et al. 2013), were found in other studies to bind G-quadruplexes, which also are in non-B DNA conformations. However, not all proteins that bind G-quadruplexes bind RNA/DNA hybrids. Most likely, proteins can distinguish between different types of non-B structures; for example, TPM4 (von Hacht et al. 2014) and BLM helicase (Li et al. 2001; Chatterjee et al. 2014) that bind G-quadruplexes did not bind to either hybrid although they are expressed in our B-cell lysates.

## Discussion

In this study, we identified more than 800 proteins that bound to RNA/DNA hybrids. R-loops are three-stranded structures that comprise an RNA/DNA hybrid and a displaced ssDNA. Here, we focused on the hybrid because the ssDNA has been the focus of many studies in the DNA repair field. We used two biotinylated hybrids corresponding to R-loops in the promoter of *BAMBI* and 3′ UTR of *DPP9* to pull down proteins that were then identified by mass spectrometry. The resulting proteins include the well-characterized RNase H1 (RNASEH1) that is known to bind to hybrids, but most of the proteins were not known to interact with hybrids. We classified these hybrid-binding proteins into those (84) that are repelled by hybrids and those (364) that are attracted to hybrids relative to the underlying dsDNA. Although the hybrid-binding proteins were identified through in vitro studies, we provide evidence that they interact with R-loops in vivo.

Cellular functions rely on proteins and their interactions with each other and with nucleic acids. Although binding does not imply function, this set of hybrid-binding proteins helps to narrow down where one should focus further investigations. Such efforts are particularly useful at the beginning of studies in which preliminary results suggest many pathways could be involved and targeted analysis may miss critical pathways. Studies are elucidating the functions of R-loops, but much remains unknown. The 803 hybrid-binding proteins described in this paper suggest that proteins involved in RNA processing from splicing to unwinding RNA are involved in hybrid-mediated regulation. RNA/DNA hybrids are also key components in DNA replication as Okazaki fragments. Molecular studies of the hybrid-binding proteins identified in this study can elucidate how proteins divide their roles (or not) between transcription and DNA replication. These hybrid-binding proteins serve as a starting point for studying how proteins recognize RNA/DNA hybrids and the functional consequences of these interactions. Delineation of how these proteins interact with RNA/DNA hybrids in transcription, DNA replication, and other cellular processes will deepen our understanding of these crucial biological pathways.

## Methods

### Cell culture

Immortalized B-cells (Coriell) were cultured to a density of 5 × 10^5^ cells/mL in RPMI 1640 supplemented with 15% fetal bovine serum, 2 mM L-glutamine, and 100 units/mL penicillin-streptomycin.

### Biotinylated RNA/DNA hybrids

Ninety-mer RNA and DNA oligos corresponding to *DPP9* 3′ UTR (Chr 19: 4,675,244–4,723,855) were synthesized by Integrated DNA Technologies (Supplemental Table S4). Oligos were dissolved in Annealing Buffer (10 mM Tris at pH 8.0; 50 mM NaCl, 1 mM EDTA). To generate the RNA/DNA hybrid, 10 µM of each oligo was mixed and heated for 5 min at 95°C and cooled down gradually to room temperature.

Six hundred-mer RNA/DNA hybrid corresponding to *BAMBI* promoter (Chr 10: 28,966,424–28,971,868) was generated as previously described (Grunseich et al. 2018). dsDNA was prepared by PCR using biotinylated primers (Supplemental Table S4). The 600-nt RNA transcript was synthesized from this *BAMBI* dsDNA template using MEGAscript T7 Transcription kit (Thermo Fisher Scientific, #AM1334). The T7 promoter sequence in dsDNA was then removed by SfcI (NEB, # R0561S) digestion. The dsDNA and the transcribed ssRNA were dissolved in 10 mM Tris HCl at pH 8.0, 1 mM EDTA, 50 mM NaCl, and incubated for 5 min at 95°C and slowly cooled down to room temperature. Reannealed dsDNA was removed by HpaII digestion (NEB, #R0171S), and the RNA/DNA hybrid was purified using agarose gel electrophoresis.

To confirm the integrity of RNA/DNA hybrids, each hybrid was digested with RNase H1 (a gift from Dr. Robert Crouch at the NIH) or RNase T1 (Thermo Fisher Scientific, #EN0541) for 1 h at 37°C, extracted with phenol/chloroform, and precipitated with ethanol. The digested hybrid was analyzed by agarose gel electrophoresis and used in protein precipitation experiments.

### Hybrid-binding protein precipitation and Western blot

Cultured B-cells were lysed in lysis buffer (20 mM Tris HCl at pH 8, 137 mM NaCl, 10% glycerol, 1% NP-40, and 2 mM EDTA) supplemented with 1× Complete protease inhibitors (Roche), 1× phosphatase inhibitors II and III (Sigma), and 0.1 unit RNase inhibitor (Thermo Fisher Scientific). Cell lysates were precleared for 2 h at 4°C using streptavidin beads (Thermo Fisher Scientific, #65305). Thirty picomoles of biotinylated RNA/DNA hybrid or dsDNA were conjugated with streptavidin beads and incubated with precleared lysates containing 240 µg total protein for 2 h to overnight at 4°C. Protein-nucleotide complexes were pulled down with streptavidin beads and washed three times in 20 mM Tris at pH 7.5, 10 mM NaCl, 0.1% Tween-20. Proteins were eluted in 1× LDS sample buffer (Thermo Fisher Scientific, #NP0007) containing 1× sample reducing reagent (Thermo Fisher Scientific, #NP0004) for 5 min at 95°C.

Hybrid-binding protein was validated by Western blot using the following antibodies: anti-NONO (Novus, #NB100-1556), anti-NPM1 (Cell Signaling, #3542), anti-RECQL (Novus, #NB100-619), and anti-GAPDH (Santa Cruz, #sc-25778).

### Mass spectrometry analysis

Liquid chromatography–tandem mass spectrometry (LC-MS/MS) analysis was performed. Each eluted sample from protein precipitation was divided into five fractionated by one-dimensional SDS-PAGE. Each fraction was digested in gel, and tryptic peptides were injected onto a UPLC Symmetry trap column (180 µm i.d. × 2 cm packed with 5 µm C18 resin; Waters). A blank gel slice was digested and injected as a background control. Tryptic peptides were separated by reversed phase HPLC on a nanocapillary analytical column (75 µm i.d. × 25 cm, 1.7 µm particle size; Waters). Eluted peptides were analyzed on a Q Exactive HF mass spectrometer (Thermo Fisher Scientific).

MS/MS spectra were searched against the UniProt human database (The UniProt Consortium 2017) with the MaxQuant 1.5.2.8 program (Cox and Mann 2008) using full tryptic specificity allowing up to two missed cleavages. Search parameters include static carboxamidomethylation of Cys, variable protein N-terminal acetylation, and variable Met oxidation. For statistical analysis, we carried out a decoy database search to determine the false discovery rate (Elias and Gygi 2010). FDR for both protein and peptide identifications was set at <1%. In the input B-cell lysate, we identified 22,440 unique peptides.

To identify proteins that specifically bind to hybrids or dsDNA in the pull-down experiments, we required at least four peptides with unique sequences from a given protein to be included. Using this criterion, 1460 and 1018 proteins were pulled down by *BAMBI* and *DPP9* hybrids, respectively, and 1092 and 995 proteins were pulled down by *BAMBI* and *DPP9* dsDNA, respectively. We focused on proteins that were reproducibly pulled down by both *BAMBI* and *DPP9* in downstream analysis. To exclude the possibility that the hybrid-binding proteins are pulled down through nonspecific binding to nucleic acids, we pre-incubated B-cell extract with biotinylated ssRNA and dsDNA sharing the same sequence with *BAMBI* hybrid and depleted nonspecific proteins using streptavidin beads. We then repeated pull down and LC-MS/MS using *BAMBI* hybrid and showed that all hybrid-binding proteins were still specifically pulled down. Fold enrichment of proteins in pull down is calculated as the ratio of MS/MS counts of a detected protein from two samples. We set the threshold of fold enrichment ≥1.2 for a protein to be considered enriched.

### RNA/DNA hybrid immunoprecipitation with qPCR (DRIP-PCR) and sequencing (DRIP-seq)

DRIP was adapted from a previous report (Skourti-Stathaki et al. 2011) with modification. Cultured B-cells (5 × 10^6^) were lysed in 400 µL cell lysis buffer (50 mM PIPES at pH 8.0, 100 mM KCl, 0.5% NP-40), and nuclei were collected by centrifugation. The nuclei pellet was resuspended in 200 µL nuclear lysis buffer (25 mM Tris HCl at pH 8.0, 1% SDS, 5 mM EDTA). Genomic DNA containing R-loops was then extracted by phenol:chloroform and precipitated by ethanol. Purified material was resuspended in 200 µL IP dilution buffer (16.7 mM Tris HCl at pH 8.0, 1 mM EDTA, 0.01% SDS, 1% Triton X-100, 167 mM NaCl) and sonicated at 4°C in Bioruptor (Diagenode) at Hi setting (30 sec on/30 sec off) for 5 min, three times, to fragments with an average size of 500 bp. Three micrograms of S9.6 monoclonal antibody (a gift from Dr. Stephen H. Leppla at NIH) or nonspecific mouse IgG (Santa Cruz, #sc-2025) was used for each immunoprecipitation. Input and precipitates were analyzed by quantitative PCR using specific primers (Supplemental Table S4).

DRIP-seq libraries were prepared from DRIP DNA and corresponding input DNA using Ovation Ultralow System (NuGen) and sequenced on an Illumina HiSeq 2500 platform. An average of 40 million 100-nt reads per sample was generated. Sequencing reads were preprocessed to remove adapter sequences from the end of reads using the program fastx_clipper from FASTX-Toolkit (http://hannonlab.cshl.edu/fastx_toolkit/). Low-quality sequences at the ends of reads represented by stretches of “#” in the quality score string in FASTQ file were also removed. Reads shorter than 35 nt after trimming were excluded from analysis. Sequencing reads were aligned to human reference (hg19) using GSNAP (version 2013-10-28) (Wu and Nacu 2010) using the following parameters: mismatches % [(read length + 2)/12-2]; mapping score R 20; soft-clipping on (-trim-mismatch-score = -3). Reads with identical sequences were compressed into one unique sequence. R-loop peaks were identified using MACS2 (Zhang et al. 2008) and required to have ≥twofold enrichment in DRIP over input. We identified 2636 R-loop peaks, among which 1438 peaks reside in 743 genes.

### SRSF1 PAR-CLIP

PAR-CLIP was performed as previously described with minor modifications (Hafner et al. 2010). Cultured human B-cells were treated with 1 mM 4-thiouridine (Sigma-Aldrich) and UV crosslinked at 312 nm for 5 min. The cells were collected, washed in 1×PBS, and fractionated into cytoplasmic and nuclear fractions. The lysate was treated with 1 unit/µL of RNase T1 for 15 min at 22°C. SRSF1 was immunoprecipitated with the anti-SRSF1 antibody (ABCAM, #ab38107). The beads with precipitates were washed three times with NP-40 lysis buffer and subsequently treated with 10 units/µL of RNase T1 for 15 min at 22°C. The beads were washed again three times in NP-40 lysis buffer and dephosphorylated with 0.5 unit/mL CIP alkaline phosphatase. The immunoprecipitated material was treated with 0.5 µCi γ-^32^P-ATP and 1 unit/µL of T4 PNK kinase for 30 min at 37°C. Beads were washed five times with PNK wash buffer (50 mM Tris HCl at pH 7.5, 50 mM NaCl, 10 mM MgCl_2_) and resuspended in 100 µL 2× sample buffer and separated on a 4%–12% SDS-PAGE and transferred to a nitrocellulose membrane. The SRSF1 ribonucleoprotein complex was visualized by autoradiography, and the band corresponding to SRSF1 was isolated. RNA was extracted by Proteinase K digestion, purified by phenol-chloroform extraction, and precipitated with three volumes of ethanol. The purified RNA from each cellular fraction was ligated with a unique 3′ adapter with Rnl2(1–249) K227Q ligase (NEB) overnight at 4°C. The RNA was loaded onto a 15% Urea-PAGE, and the ligated RNA cut out and extracted from the gel with 400 µL 0.3M NaCl for 45 min at 60°C with vigorous shaking. The gel pieces were filtered away and RNA in the flow-through precipitated with three volumes of ethanol. The RNA pellet was dissolved in water and ligated with 5′ adapter using Rnl1 ligase (NEB) for 1 h at 37°C. The RNA was loaded onto a 12% Urea-PAGE, and the ligated RNA cut out and extracted from the gel with 400 µL 0.3 M NaCl for 45 min at 60°C with vigorous shaking. The gel pieces were filtered out and RNA in the flow-through precipitated with three volumes of ethanol. The RNA was reverse transcribed using SuperScript III reverse transcriptase (Thermo Fisher Scientific) with 3′ RT primer for 2 h at 50°C, according to the manufacturer's instructions. Next, the generated cDNA was PCR amplified using Taq DNA polymerase (Thermo Fisher Scientific). The primers used for PAR-CLIP are listed in Supplemental Table S4. The PCR band corresponding to the correct size of amplification (143–153 bp) was purified using a 3% PippinPrep gel according to the manufacturer's instructions and quantified. PAR-CLIP cDNA libraries were sequenced on an Illumina HiSeq 3000 instrument. Clusters of overlapping reads uniquely mapped to the human genome hg19 were generated using the PARalyzer software (Corcoran et al. 2011), allowing for one mismatch and otherwise default settings. Clusters were annotated against the following GENCODE gtf file: GENCODE.v19.chr_patch_hapl_scaff.annotation.gtf (http://www.gencodegenes.org) (Harrow et al. 2012). The hg19 assembly was used. The main differences between hg19 and the more current GRCh38 is that GRCh38 contains more alternatively spliced sequences, centromeric regions, and the mitochondria genome. Because these sequences were not the focus of our study, the use of hg19 is unlikely to have affected our conclusions.

### Reverse protein-RNA immunoprecipitation and sequencing

Reverse immunoprecipitation was carried out using Magna RNA-Binding Protein Immunoprecipitation Kit (Millipore) following the manufacturer's protocol. Briefly, for each immunoprecipitation reaction, 2 × 10^7^ cultured human B-cells or primary skin fibroblasts were harvested and lysed in 100 µL lysis Buffer with protease and RNase inhibitors. Five micrograms of anti-SRSF1 antibody (ABCAM, #ab38107), anti-FUS antibody (Novus, #NB100-561), and anti-DDX1 (ABCAM, #ab70252), negative control IgG (Millipore, #12-371 for mouse IgG; #12-370 for rabbit IgG) were conjugated to Magnetic Protein A/G beads. One hundred microliters of cell lysate was added into 900 µL Immunoprecipitation Buffer with RNase inhibitor and incubated with 50 µL beads-antibody complex overnight at 4°C. Bead-bound immunoprecipitates were then washed six times using cold Wash Buffer with RNase inhibitor and incubated with protease K in the presence of 1% SDS for 30 min at 55°C. RNA and DNA were then extracted from supernatants using phenol:chloroform:isoamyl alcohol and precipitated using ethanol. Precipitated RNA was digested by DNase I (DNA-free kit, Ambion). cDNA was synthesized using random hexamer primer by TaqMan Reverse Transcription Reagent kit (Applied Biosystems). Quantitative PCR was carried out to quantify cDNA and DNA with primers annealing to *BAMBI* and *DPP9* hybrids using Power SYBR Green PCR Master Mix (Thermo Fisher Scientific, #4367659). Primer sequences are listed in Supplemental Table S4. RNA from anti-SRSF1 immunoprecipitate and input RNA were prepared into RNA-seq libraries using Illumina TruSeq Stranded Total RNA Library Prep kit (Illumina, #20020596) and sequenced on HiSeq 2500. Sequencing reads were preprocessed and aligned as described above. Enrichment of transcripts in the immunoprecipitate was analyzed using the program described by Antanaviciute et al. (2017). Transcripts with fold enrichment >2 by anti-SRSF1 antibody are considered SRSF1-binding targets.

### Biolayer interferometry

Analysis of dsDNA or RNA/DNA hybrid binding to candidate proteins was carried out using the Octet RED96 system (ForteBio) with sensor detection of the change in wavelength (nm shift). Purified candidate proteins DDX5 (Abnova, #TP300371), NONO (Abnova, #TP326567), SUPT5H (Abnova, #TP326321), and human RNase H1 (RNASEH1; a gift from Dr. Robert Crouch at the NIH) were evaluated. Biotinylated dsDNA or RNA/DNA hybrid at concentrations of 5 nM was immobilized onto a Streptavidin-SA biosensor. The biotinylated *DPP9* dsDNA 90-mer was generated as described above. dsDNA and RNA/DNA hybrid were loaded onto the sensors until saturation. The nucleotide-labeled sensors were then washed with buffer, followed by addition of DDX5 at concentrations of 2 and 8 µg/mL, NONO at concentrations of 1 and 4 µg/mL, SUPT5H at concentrations of 2 and 8 µg/mL, and RNase H1 (RNASEH1) at 3.8 and 19 nM. All reactions were tested in TBS buffer (10 mM Tris at pH 7.4, 68 mM NaCl, 0.02% Tween-20). A reference sample of buffer and protein alone did not show any signal drift. Association and dissociation were monitored for 10 min each. All experiments were conducted in the Octet instrument with agitation at 1000 rpm.

### Immunofluorescence

Fibroblasts were fixed with 4% paraformaldehyde for 15 min at room temperature, then washed three times with phosphate-buffered saline (PBS). Slides were then placed in blocking solution (5% normal goat serum, 0.3% Triton X-100 in PBS) for 1 h at room temperature. Primary antibody staining was done overnight at 4°C in PBS with 1% bovine serum albumin and 0.3% Triton X-100 using 1:500 S9.6 antibody, 1:500 DDX18 antibody (ABCAM, #ab128197), or 1:500 nucleolin antibody (ABCAM, #ab22758). Slides were then washed three times with PBS for 5 min each, incubated with 1:500 secondary antibody (Invitrogen, #A-31572 for anti-rabbit and #A-11001 for anti-mouse) for 2 h at room temperature in the dark, and then washed three times with PBS for 5 min each before DAPI nuclear staining. Imaging was performed with a Leica DMI 6000CS laser confocal microscope with a Leica HCX PL APO 63× oil objective.

#### Intrinsically disordered region

IUPred (Dosztányi et al. 2005) was used to predict disordered regions in the protein. The 354 proteins that we included as containing disordered regions are those with at least 30% of the proteins with IUPred score >0.4 (Hentze et al. 2018). In “long” (global) disorder mode, a sequential neighborhood of 100 residues is considered in calculating the score, whereas in “short” (local) disorder mode, a sequential neighborhood of 25 residues is considered.

## Data access

The PAR-CLIP, RNA-IP-seq, and DRIP-seq data from this study have been submitted to the NCBI Gene Expression Omnibus (GEO; https://www.ncbi.nlm.nih.gov/geo/) under accession number GSE117671. The mass spectrometry data from this study have been submitted to the PeptideAtlas database (http://www.peptideatlas.org/) with the identifier PASS01169.

## Supplementary Material

Supplemental Material
