# Supplementary material for: Human proteins that interact with RNA/DNA hybrids
Source: Genome Res. 2018 Sep;28(9):1405–14. doi: 10.1101/gr.237362.118 (PMC6120628; doi:10.1101/gr.237362.118)
Supplement: Supplemental Material [file supp_28_9_1405__index.html]

Human proteins that interact with RNA/DNA hybrids — Supplemental Material 

# Human proteins that interact with RNA/DNA hybrids

## Supplemental Material

- Supplemental\_Table\_S1.xlsx
- Supplemental\_Table\_S2.xlsx
- Supplemental\_Table\_S3.xlsx
- Supplemental\_Table\_S4.xlsx
